# Effect of ART on poly functional profile of CD8 responses to *Gag* and *Nef* antigen in HIV infected Indian individuals

**DOI:** 10.1186/1471-2334-14-S3-O7

**Published:** 2014-05-27

**Authors:** Sanjay Mendiratta, Ravinder Singh, Madhu Vajpayee

**Affiliations:** 1 All India Institute of Medical Sciences, New Delhi, India; 2 National Institute of Biologicals, Noida, India

## Background

Poly functional CD8+ T cells have been described most competent in controlling viral replication in late stage of HIV infection.

## Methods

Multiparametric flow cytometry and intracellular cytokine staining was performed to study the effect of HAART on poly functional responses of CD8+ T lymphocytes to HIV *Gag* and *Nef* peptides and polyclonal stimuli in 40 ART naïve and 10 ART treated HIV infected individuals to compare and examine the degree of immune restoration after ART. We analyzed the CD8+ T cell responses (IFN-γ, IL-2, TNF-α, CD107a) and generated fifteen unique subsets using Boolean gating (Flowjo, SPICE-PESTLE software).

## Results

Bulk response of HIV-1–specific CD8+ T cells directed against the identified immunodominant peptides in the treated group decreased (average ± SD: *Gag*: 2.1% ± 2.1% to 1.17% ± 1.17%, *p* = 0.14; *Nef*: 2.95 ± 3.23 to 1.33% ± 1.04%, *p* = 0.14; Polyclonal Activator: 15.16% ± 8.74% to 5.93% ± 8.29%, *p* = 0.007). The responses were predominantly mono functional in ART naïve individuals, as also shown by their level of responses and contribution towards global responses. Dual function responses were increased in ART treated individuals as compared to naïve study subjects.

We also observed slight increase in IL2 and TNFα secreting CD8+ T cell subsets in ART treated individuals.

## Conclusion

Our data indicates that the functional impairment of antigen specific CD8+ T cells in HIV infection is dynamic and can be partially restored with ART. However the data needs to be substantiated on large sample size.

